# Aroma-based discrimination of Egyptian versus Indian guava fruits and in response to probiotics as analyzed via SPME/GC–MS and chemometric tools

**DOI:** 10.1038/s41598-023-45686-z

**Published:** 2023-10-27

**Authors:** Islam M. Kamal, Ahmed Zayed, Tarek F. Eissa, Mohamed A. Farag

**Affiliations:** 1 Microbiology and Immunology Department, Faculty of Pharmacy, Cairo University, Kasr El Aini St., Cairo, 11562 Egypt; 2 Pharmacognosy Department, College of Pharmacy, Tanta University, Elguish Street (Medical Campus), Tanta, 31527 Egypt; 3 Faculty of Biotechnology, October University for Modern Sciences and Arts (MSA), Giza, 12451 Egypt; 4 Pharmacognosy Department, College of Pharmacy, Cairo University, Kasr El Aini St., 11562, Cairo, Egypt

**Keywords:** Biotechnology, Chemical biology, Microbiology, Plant sciences

## Abstract

Guava tree (*Psidium guajava* L., Myrtaceae) is an economic grown worldwide, particularly in tropical and subtropical regions. Guavas encompass numerous cultivars (cvs.) that were discriminated in previous studies based on leaf morphological features and profile of volatile organic compounds (VOCs). Nevertheless, fruit VOCs have also shown outstanding potential for discrimination of other plant taxa, which has not been utilized in guava. Hence, the current study investigates the various guava cvs. harvested from India and Egypt. A total of 5 samples were analyzed by solid phase microextraction coupled to gas chromatography/mass spectrometry. Results led to the detection of 42 VOCs belonging to aldehydes, alcohols, esters, ketones, aliphatic and aromatic hydrocarbons, in addition to monoterpene and sesquiterpene hydrocarbons. Butylated hydroxytoluene and *β*-caryophyllene were predominant reaching 77% and 41% in Egyptian and Indian guava, respectively. The impact of probiotic fermentation, i.e., *Lactobacillus acidophilus* and *L. plantarum* on aroma profile was not significantly different (*p* > 0.05). Multivariate data analyses were further applied for samples classification and markers determination, including principal component analysis (PCA) and orthogonal partial least squares discriminant analysis (OPLS-DA). PCA score plot showed clear segregation of Egyptian from Indian specimens, whereas OPLS-DA revealed that *β*-caryophyllene was associated with white fruit versus 3-butenyl isothiocyanate and muurolol in red fruit type in the case of Indian guava. The richness of Egyptian guava in butylated hydroxytoluene in addition to the presence of vitamin C may potentiate its antioxidant activity, to be followed in subsequent studies regarding its health effects.

## Introduction

With around 266 species, the genus *Psidium* is recognized as well-known food source for animals, and humans ascribed to their edible fruits^1^. Among them, guava tree (*Psidium guajava* L., family Myrtaceae) is a tropical crop that grows natively in warm and dry climates, specifically Central America. Currently, about 60 countries in tropical and sub-tropical regions are major producers reaching more than 36 million tons in 2017 and increased to about 55.85 million tons in 2019^2^ and estimates of 86.74 billion in 2022, including India, China, Egypt, and other countries in South and Central America (e.g., Mexico and Brazil)^3–6^. The interest in guava cultivation has been reported similar to mango (*Mangifera indica* L.) owing to its nutritive value in addition to several health benefits of its different parts such as leaf, root, bark, and fruit^5,7^. The ethnobotanical uses of various extracts include anti-microbial, anti-diabetic to anti-viral effects^8,9^.

Postharvest processing results in a number of byproducts, including seeds, peels, pulp remain and leaves. Particularly, leaves are rich in phenolic secondary metabolites (e.g., flavonoids, phenolic acids, and essential oils) with potential antioxidant, anti-inflammatory, anti-bacterial, and antidiabetic effects, and therefore, they constitute a main part in various marketed preparations, including cosmetics, medicines (e.g., antispasmodic and anticough), foods, and beverages^7,10–12^. Likewise, fruits are rich in phytochemicals (e.g., essential oil, anthocyanins, carotenoids, and polyphenols), vitamin A and C, and minerals. Nevertheless, the essential oil profile of leaf is different from that of fruit. For instance, El-Ahmady, et al. found that *β*-caryophyllene and limonene were predominant in essential oil derived from fruit in Egypt, while *β*-caryophyllene and selin-7(11)-en-4*α*-ol predominated leaf oil composition prepared using hydro-distillation^13^. In other studies, cinnamyl alcohol, ethyl benzoate, *β*-caryophyllene, (*E*)-3-hexenyl acetate, and *α*-bisabolene were identified as major constituents in fruit cvs. grown in Thailand using solid phase microextraction (SPME) coupled to gas chromatography (GC)/mass spectrometry (MS). Also, volatile organic compounds (VOCs) such as (*E*)-*β*-ionone, ethyl hexanoate, ethyl butanoate, hexanal, (*Z*)-3-hexenal, hexyl acetate, (*E*)-2-hexenal, and limonene were recognized as the most typical guava aroma of Red Suprema cultivar (cv.)^14^. The presence of such fragrant compounds results in a unique taste and flavor of the fruits^15^. In addition, presence of dietary fibers (e.g., cellulose, hemicellulose, and pectin) up to 43.21% provides several nutritional benefits of food products contained either fresh or processed fruits (e.g., jams, juices, and desserts)^4,11^.

Guava is represented by various varieties which have attracted a special interest for their discrimination based on leaves’ morphological features^16^ and volatile composition^17,18^. Also, two common varieties of guava are reported according to flesh pulp’s color, viz. red (*P. guajava* var. *pomifera*) and white (*P. guajava* var. *pyrifera*)^19^. Phytochemical differences between them were observed in vitamin C and total phenolics, with red variety more enriched^20,21^. Fruits aroma has shown its potential to discriminate between numerous plant cvs. such as Egyptian mango^22^, strawberry^23^, and apples^24^. However, discrimination based on fruits’ aroma has not yet been performed for guava especially from different origins. Moreover, previous reports have demonstrated that aroma profile of foods to be affected by probiotics that inhabit human gut, e.g., mango fruits^22^, and roselle flower^25^ especially if incorporated in dairy products such as yogurt, etc.

Using the same approach applied previously for mango fruits aroma profiling, i.e., SPME coupled to GC–MS^22^, we extend herein to analyze guava fruits in context to their aroma composition from different origins that is India and Egypt (tropical vs. subtropical) at different seasons from different varieties commonly grown in these habitats. Further, the present study investigated another process, i.e., inoculation of probiotic bacteria, that is used commonly in fermented dairy products. *Lactobacillus acidophilus* and *Lactiplantibacillus plantarum* (formerly *Lactobacillus plantarum*) were chosen as representatives of probiotics^22,26^.

Multivariate data analyses (MVDA) including non-supervised [e.g., principal component analysis (PCA)] in addition to supervised [e.g., orthogonal partial least squares discriminant analysis (OPLS-DA)], are considered an integral part of plant metabolomics, especially upon handling of huge dataset for identification of similarities, hidden pattern, and associated markers^27,28^. Therefore, the current study integrates the results of aroma profile in guava cvs. from different origins, and in response to probiotics.

## Material and methods

### Plant materials

A total of five guava fruit cvs. were harvested either in winter (E-RG-W, I-RG-W, and I-RG-R) or summer (E-SFG and E-RG), from Egypt (3 cvs.; E-SFG, E-RG-W. and E-RG) and India (2 cvs; I-RG-R and I-RG-W.), Table 1. The Egyptian cvs. collected from Rashid Adico region, North Egypt (31° 24′ 16″ N 30° 24′ 59″ E) was characterized by their yellowish white fleshy fruit pulp, 2 of which possessed rough skin, i.e., E-RG-W and E-RG, whereas one showed smooth surface (E-SFG). In case of Indian guava cvs. collected from Hisar fields (29° 09′ N 75° 42′ E), both had rough skin, while the fleshy pulp was either red (I-RG-R) or white (I-RG-W). The fruits were directly washed and sliced into small pieces before being stored at − 20 °C till further aroma analysis. All experimental procedures were carried out in accordance with the relevant laws and guidelines, including the appropriate permissions for the collection of plant specimens.

**Table 1:** A list of investigated guava fruit accessions in the current study, their attributes, and codes.

Sample code	Color of the pulp	Skin texture	Season of harvesting	Location of harvesting
E-SFG	Yellowish white	Soft	Summer	Rashid Adico, Egypt
E-RG-W	Yellowish white	Rough	Winter	
E-RG	Yellowish white	Rough	Summer	
I-RG-R	Red	Rough	Winter	Hisar, India
I-RG-W	White	Rough	Winter	

### Chemicals and fibers

Chemicals and standards were purchased from Sigma-Aldrich® (St. Louis, MO, USA). In addition, divinylbenzene/carboxen/polydimethylsiloxane (DVB/CAR/PDMS 50/30 um) coated SPME StableFlex fibers were purchased from Supelco® (Oakville, ON, Canada). Prior to volatiles collection, the fibers were conditioned at 250 °C for 5 min following supplier’s recommendations.

### Headspace volatile analysis of guava fruits

Headspace (HS)-SPME was utilized for volatiles collection prior to analysis by GC–MS. Three biological replicates (n = 3) from each guava fruit cv. were included, being able to assess biological variance. Volatiles analysis was carried out following our previous protocol reported in mango fruits^22^ with slight modifications. More information about volatiles collection, optimization, and analysis using SPME coupled to GC/MS can be found in our previous reports^29^. In brief, each cv. slices were transferred to in a screw-capped 1.5 mL-vial, while 1 μL of aqueous (*Z*)-3-hexenyl acetate was added as an internal standard at a final concentration of 10 μg^30^. Afterwards, the needle containing the SPME fiber was manually introduced above the sample and heated at 50 °C for 15 min for volatiles adsorption. Then, the fiber was withdrawn, and the needle was directly introduced to the GC injection port of a Shimadzu GC-17A gas chromatograph equipped with DB-5 column (30 mL, 0.25 mm × 0.25 um film thickness; Supelco^®^), while the mass spectrometer was Shimadzu QP5050A. The GC was set at a specified temperature gradient used previously also and the mass spectrometer was operated in EI mode at 70 eV for compounds scanning in the range of *m/z* 40–500^22^.

### Probiotics treatment

Following the procedure performed for roselle and mango probiotic treatment by our group^22,25^, Egyptian guava fruit cv. with soft skin and yellowish white fleshy pulp (E-SFG) was chosen as a representative for other accessions. Freshly prepared guava juice was treated with representative probiotic bacteria, including *L. acidophilus* and *L. plantarum*. Prior to bacterial inoculation, guava juices were diluted with distilled water at a ratio of 1:2, autoclaved shortly for 5 min, and then, rapidly cooled at 4 °C. Afterwards, bacterial inoculum in 2 mL PBS (pH 7.4) at 10^9^ CFU/mL (OD_600_ = 0.36) were introduced to 100 mL of previously prepared guava juice at initial pH = 5.0 ± 0.2.

Aliquots of 15 mL were sampled to cotton plugged glass tubes at regular time intervals; 0, 24 and 48 h, whereas pH, viable count (CFU/mL), and volatiles were examined as previously described in volatiles analysis by HS-SPME coupled with GC–MS using the same protocol. Three biological replicates were used (n = 3), except that of the 48 h which included 4 replicates (n = 4).

### Volatiles identification and data extraction from GC–MS

Comparison with Kovat index (RI) relative to *n*-alkanes (C_6_–C_20_), mass matching to NIST database, WILEY Library database and standards (whenever available) were applied for volatile peaks identification. AMDIS software (www.amdis.net) was applied firstly for peaks deconvolution prior to spectral matching. Aside from that, MS-Dial tool (http://prime.psc.riken.jp/compms/msdial/main.html) was used for data extraction and volatile abundance data preparation for the subsequent MVDA. The various dataset was statistically analyzed by PCA, HCA, and OPLS-DA using SIMCA-P version 14.1 software package (Umetrics, Umea Sweden). All variables were mean centered, and Pareto scaled^31^. Due to the limited number of samples, the whole dataset was used as a training set. Then, sevenfold cross-validation was applied in order to determine the optimum number of principal components, i.e., the latent variables, and test the model’s predictive ability via calculating the Q2. Furthermore, permutation tests and *p* value determination have been implemented as extra procedures to ensure the validity of the developed models.

## Results and discussion

### Volatiles identification and classification

The cvs. of guava were chosen based on different factors to include geographical origin i.e., tropical versus subtropical, morphological features i.e., fruit pulp color and skin texture. A list of the investigated cvs. and their codes were summarized in Table 1. DVB/CAR/PDMS coated fiber was chosen for analysis based on its efficiency, universal adsorption power, and sensitivity shown in our previous similar work on food aroma analysis compared to polydimethylsiloxane (PDMS) analogue^30,32^.

**Figure 1:**
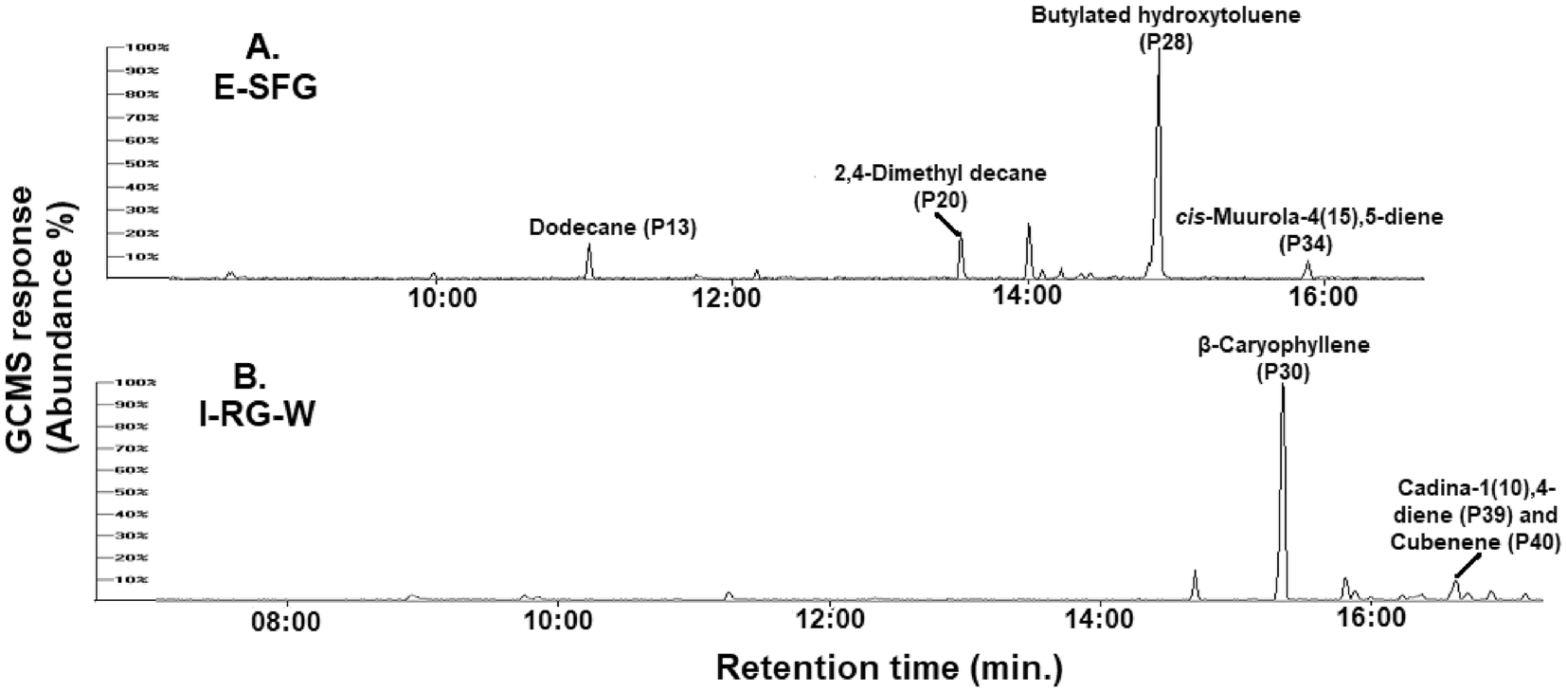
Representative GC–MS chromatograms of 2 guava fruits for their volatiles analysed by SPME coupled with GC–MS; (**A**) Egyptian guava fruit with soft yellowish white pulp (E-SFG) and (**B**) Indian guava fruit with rough white pulp (I-RG-W).

The GC–MS total ion chromatograms showed a total of 42 peaks. Figure 1 showed representative GC–MS chromatograms of two guava fruit samples, including Egyptian guava fruit with soft yellowish white pulp (E-SFG) and Indian guava fruit with rough white pulp (I-RG-W). The two chromatograms looked qualitatively different regarding the number and major peaks. These results indicated the effect of cvs. and or growing habitat on guava fruits VOCs profile. For more information and overall overview, the five chromatograms are shown in Fig. S1.

A list of identified compounds and their percentages of relative abundances was summarized in Table 2. It is noteworthy that the current study could identify more VOCs compared with only 24 compounds in white flesh guava fruit cultivated in Reunion Island, despite that both studies applied the same technique for volatiles collection, i.e., SPME^33^ likely attributed to data processing using AMDIS software.

**Table 2:** Relative abundances (%) of identified volatiles in different guava fruits cultivars as determined via SPME coupled with GC–MS.

Peak #	Average Rt (min)	Calculated KI	Theoretical Kovat retention index (KI)	Metabolite	Class	Relative abundance (%) ± St. dev				
						E-SFG	E-RG-W	E-RG	I-RG-R	I-RG-W
1	5.494	801	771	Hexanal	Aldehydes	0.06 ± 0.05	0.02 ± 0.02	0.02 ± 0.01	1.79 ± 0.9	3.12 ± 0.31
2	6.622	884	823	(*E*)-2-Hexenal*		0.00 ± 0.0	0.05 ± 0.05	0.03 ± 0.0	1.410.51	1.80 ± 0.27
3	6.642	886	860	2-Hexenal isomer		0.03 ± 0.02	0.03 ± 0.03	0.00 ± 0.0	0.33 ± 0.09	0.25 ± 0.13
12	10.882	1173	1128	Nonanal		0.02 ± 0.02	0.05 ± 0.0	0.00 ± 0.0	0.34 ± 0.1	0.29 ± 0.05
Total aldehyde relative abundances (%)						**0.11 ± 0.1**	**0.15 ± 0.1**	**0.05 ± 0.01**	**3.86 ± 1.59**	**5.46 ± 0.72**
4	6.703	890	847	3-Hexen-1-ol*	Alcohols	0.03 ± 0.04	0.02 ± 0.2	0.01 ± 0.01	8.31 ± 2.22	8.40 ± 2.71
6	6.775	896	838	Hexen-1-ol isomer		0.00 ± 0.0	0.02 ± 0.02	0.02 ± 0.0	1.59 ± 0.43	1.81 ± 0.71
32	15.874	1604	1578	Globulol		0.06 ± 0.07	0.00 ± 0.0	0.04 ± 0.02	2.41 ± 0.28	3.19 ± 0.4
38	16.614	1654	1628	Muurolol		0.05 ± 0.05	0.05 ± 0.01	0.02 ± 0.02	6.89 ± 0.93	2.38 ± 0.07
Total alcohol relative abundances (%)						**0.14 ± 0.15**	**0.09 ± 0.05**	**0.08 ± 0.05**	**19.20 ± 3.86**	**15.78 ± 3.88**
5	6.71	892	612	2,3-Dimethyl-1,3-butadiene	Aliphatic hydrocarbons	0.01 ± 0.02	0.02 ± 0.03	0.02 ± 0.0	3.87 ± 2.17	3.29 ± 1.52
13	11.056	1186	209.9	Dodecane		9.76 ± 3.94	10.19 ± 4.95	1.26 ± 0.44	0.00 ± 0.0	0.00 ± 0.0
20	13.569	1398	1115	2,4-Dimethyl decane		16.50 ± 2.69	19.89 ± 1.37	6.15 ± 1.51	0.00 ± 0.0	0.00 ± 0.0
33	15.883	1604	–	Unknown		0.01 ± 0.01	0.00 ± 0.0	0.01 ± 0.01	4.30 ± 0.81	3.64 ± 0.06
35	16.004	1613	1600	Hexadecane*		0.02 ± 0.03	0.02 ± 0.02	0.00 ± 0.0	0.48 ± 0.16	0.33 ± 0.04
Total aliphatic hydrocarbon relative abundances (%)						**26.30 ± 6.69**	**30.13 ± 6.37**	**7.44 ± 1.97**	**8.65 ± 3.14**	**7.26 ± 1.62**
7	8.087	978	930	*α*-Pinene	Monoterpene hydrocarbons	0.02 ± 0.01	0.02 ± 0.02	0.01 ± 0.01	0.60 ± 0.16	0.06 ± 0.02
8	8.635	1012	981	*β*-Myrcene*		1.14 ± 0.73	3.49 ± 1.13	1.73 ± 1.72	0.00 ± 0.0	0.00 ± 0.0
11	9.747	1088	1020	Limonene		0.02 ± 0.02	0.06 ± 0.06	0.02 ± 0.0	0.90 ± 0.37	1.46 ± 0.63
Total monoterpene hydrocarbon relative abundances (%)						**1.17 ± 0.76**	**3.57 ± 1.21**	**1.76 ± 1.73**	**1.50 ± 0.53**	**1.52 ± 0.65**
9	8.917	1031	964	3-Butenyl isothiocyanate	Sulphur compounds	0.00 ± 0.0	0.05 ± 0.01	0.02 ± 0.0	8.94 ± 4.46	5.04 ± 0.99
15	11.253	1202	1055	2-(Allylthio) acetonitrile		0.02 ± 0.02	0.06 ± 0.06	0.02 ± 0.02	5.09 ± 1.07	3.73 ± 0.36
Total sulphur compounds’ relative abundances (%)						**0.02 ± 0.02**	**0.11 ± 0.07**	**0.04 ± 0.02**	**14.03 ± 5.53**	**8.77 ± 1.36**
10	8.992	1037	960	6-Methyl-5-hepten-2-one	Ketones/Esters	0.04 ± 0.02	0.04 ± 0.04	0.01 ± 0.01	1.92 ± 0.16	0.59 ± 0.15
26	14.587	1493	–	Unknown		0.09 ± 0.05	0.05 ± 0.02	0.05 ± 0.02	0.02 ± 0.01	0.03 ± 0.03
14	11.154	1194	1039	2,2,5-trimethyl-3,4-Hexanedione		0.09 ± 0.01	0.08 ± 0.08	0.16 ± 0.05	0.19 ± 0.12	0.49 ± 0.19
18	13.25	1370	1322	*α*-Terpinyl acetate*		0.01 ± 0.02	0.04 ± 0.01	0.02 ± 0.0	0.17 ± 0.05	0.03 ± 0.02
Total Ketone/Ester relative abundances (%)						**0.23 ± 0.11**	**0.22 ± 0.15**	**0.24 ± 0.07**	**2.30 ± 0.33**	**1.15 ± 0.39**
16	11.892	1254	–	Unknown	Nitrogenous compounds	0.00 ± 0.0	0.02 ± 0.02	0.02 ± 0.0	0.15 ± 0.09	0.16 ± 0.0
17	12.887	1338	1198	3-Phenyl propiononitrile		0.02 ± 0.02	0.05 ± 0.05	0.01 ± 0.01	1.32 ± 0.15	0.82 ± 0.15
Total nitrogenous compounds’ relative abundances (%)						**0.02 ± 0.02**	**0.07 ± 0.07**	**0.03 ± 0.01**	**1.47 ± 0.24**	**0.98 ± 0.24**
19	13.521	1395	1440	*cis*-Muurola-3,5-diene	Sesquiterpene hydrocarbons	0.00 ± 0.0	0.00 ± 0.0	0.00 ± 0.0	0.02 ± 0.02	0.01 ± 0.01
21	13.646	1406	1375	*β*-Elemene*		0.18 ± 0.09	9.84 ± 9.4	0.25 ± 0.06	0.00 ± 0.0	0.00 ± 0.0
22	14.092	1446	–	Unknown		2.16 ± 1.83	2.95 ± 0.75	0.72 ± 0.68	0.00 ± 0.0	0.00 ± 0.0
23	14.172	1454	1419	Isocaryophyllene		0.36 ± 0.06	0.20 ± 0.12	0.44 ± 0.43	0.01 ± 0.01	0.02 ± 0.01
24	14.222	1459	–	Unknown		1.77 ± 0.49	1.65 ± 1.53	0.39 ± 0.03	0.00 ± 0.0	0.00 ± 0.0
25	14.321	1468	1455	Aromandendrene		1.92 ± 0.58	4.14 ± 1.43	2.89 ± 0.35	0.00 ± 0.0	0.00 ± 0.0
27	14.686	1502	1376	Copaene		0.03 ± 0.02	0.02 ± 0.02	0.01 ± 0.01	4.43 ± 0.83	3.56 ± 0.31
29	15.196	1546	1547	Nerolidol		0.07 ± 0.02	0.17 ± 0.1	0.10 ± 0.06	0.00 ± 0.0	0.00 ± 0.31
30	15.567	1578	1421	*β*-Caryophyllene		0.05 ± 0.06	0.12 ± 0.07	0.01 ± 0.01	34.56 ± 7.79	40.85 ± 5.79
31	15.807	1599	1454	α-Humulene*		0.01 ± 0.01	0.00 ± 0.0	0.01 ± 0.01	1.24 ± 0.28	0.06 ± 0.04
34	15.992	1612	1490	*cis*-Muurola-4(15),5-diene		8.25 ± 1.01	3.01 ± 2.71	7.27 ± 0.07	0.00 ± 0.0	0.00 ± 0.0
36	16.225	1627	1458	*cis*-*α*-Bisabolene		1.88 ± 0.16	6.16 ± 5.37	0.78 ± 0.03	0.00 ± 0.0	0.00 ± 0.0
37	16.364	1637	1509	*β*-Bisabolene		0.01 ± 0.01	0.02 ± 0.02	0.00 ± 0.0	1.30 ± 0.15	0.34 ± 0.08
39	16.701	1660	1514	Cadina-1(10),4-diene		0.00 ± 0.0	0.00 ± 0.0	0.00 ± 0.0	3.76 ± 0.86	5.14 ± 0.92
40	16.879	1671	1345	Cubenene		0.00 ± 0.0	0.00 ± 0.0	0.00 ± 0.0	1.57 ± 0.23	4.71 ± 0.43
41	17.13	1688	1448	*β*-Farnesene		0.05 ± 0.02	0.00 ± 0.0	0.00 ± 0.0	1.13 ± 0.24	2.15 ± 0.21
42	18.467	1756	–	Unknown		0.05 ± 0.03	0.08 ± 0.02	0.02 ± 0.02	0.93 ± 0.16	2.23 ± 0.3
Total sesquiterpene hydrocarbon relative abundances (%)						**16.76 ± 4.4**	**28.35 ± 21.54**	**12.88 ± 1.74**	**48.95 ± 10.57**	**59.05 ± 8.11**
28	14.881	1518	1488	Butylated hydroxytoluene	Phenols	55.09 ± 8.67	37.23 ± 5.47	77.15 ± 0.98	0.03 ± 0.02	0.04 ± 0.02

Explanation of samples code is shown in Table 1. The values are expressed as average ± st. dev. (n = 3).

–: Not available.

*: Compounds identified with the aid of a reference standard.

The identified compounds were categorized according to their classes, where sesquiterpene hydrocarbons were the most representative by17 compounds. Other classes included aliphatic hydrocarbons (5), ketones/esters (4), alcohols (4), aldehydes (4), monoterpene hydrocarbons (3), sulfur compounds (2), nitrogenous compounds (2), and phenol (1). The predominance of sesquiterpene and monoterpene hydrocarbons is typical for volatile constituents of guava fruits, especially that showing the chemical skeleton-types of bisabolane, caryophyllane, cadinane, and aromadendrane for sesquiterpene hydrocarbons and *p*-menthane and pinane for monoterpene hydrocarbons^1,34^, Table 2.

**Figure 2:**
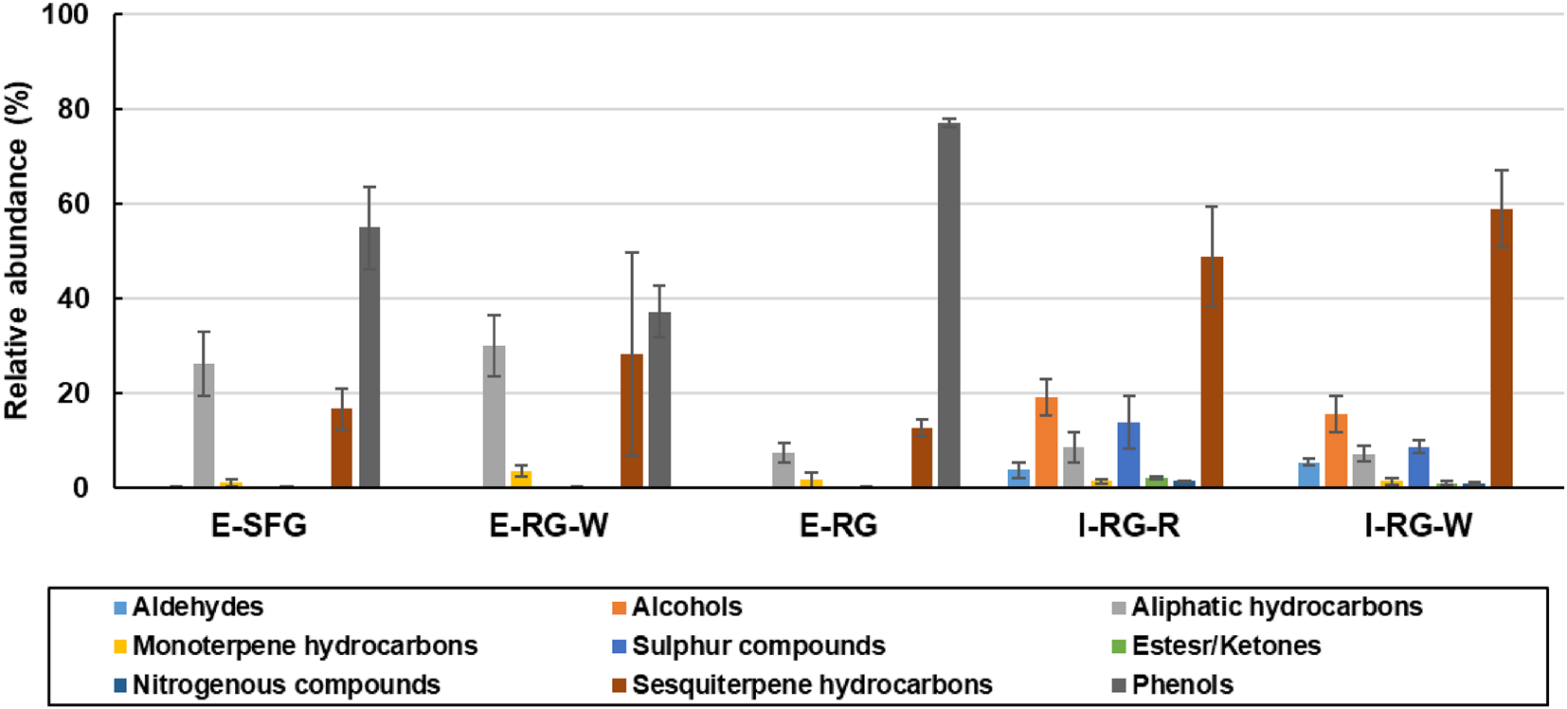
Relative abundance (%) of volatile phytochemical classes per guava fruit specimens. Mostly, sesquiterpene hydrocarbons and phenols are the major constituents in Indian and Egyptian cultivars, respectively. The sample codes follow that listed in Table 1.

Asides, the total relative abundances (%) of each phytochemical class per sample of guava fruit is represented in Fig. 2. Sesquiterpene hydrocarbons and phenols were the major constituents in Indian and Egyptian cvs. reaching their maximum abundances at 59% and 77% in I-RG-W and E-RG, respectively. Variation and main constituents among each class of VOCs will be discussed in the following subsections.

### Sesquiterpene hydrocarbons

With 17 peaks, sesquiterpene hydrocarbons were the most representative class of volatiles among other classes to account for 13–59% of guava cvs. aroma, with E-RG being the least versus I-RG-W most rich, Table 2. The other Indian cv., i.e., (I-RG-R) was also enriched in sesquiterpene hydrocarbons at 49%. The higher abundance was mostly associated with *β*-caryophyllene (P30) to amount for 41% and 35% in I-RG-W and I-RG-R, respectively. Other major, but less abundant volatiles included copaene (P27), cadina-1(10),4-diene (P39), cubenene (P40), and *β*-farnesene (P41).

In contrast, the total abundance of sesquiterpene hydrocarbons was less in Egyptian cvs. accounting for 17%, 28%, and 13% in E-SFG, E-RG-W, and E-RG, respectively. It is noteworthy to report that *β*-elemene (P21), *cis*-*α*-bisabolene (P36), and aromandendrene (P25) were major constituents detected at 9.8, 6.2, and 4.1%, respectively. These results seem to be related to the season of harvesting, indicating the effect of winter for increase in sesquiterpene hydrocarbons production and has yet to be tested by analyzing other accessions to be conclusive from other origins. *cis*-Muurola-4(15),5-diene (P34) was the most abundant in E-SFG and E-RG accounting for 8.3 and 7.3%. respectively. Additionally, *β*-caryophyllene (P30) was detected at trace levels 0.01–0.1% in the three Egyptian cvs. and opposite to previous results reporting *β*-caryophyllene as major VOCs in the fruit oil prepared using hydro-distillation^13^. Such discrepancy may be attributed to the use of other methods for volatiles' extraction compared to the relatively cold method used in SPME for volatiles collection. *β*-caryophyllene exhibits a strong wooden odor being incorporated in cosmetic and food additives as an approved flavoring agent by the Food and Drug Administration (FDA) and the European Food Safety Authority (EFSA)^35^.

### Phenols

Paniandy, et al. reported on phenols in guava fruit using HS-SPME in comparison with hydrodistillation method^33^. However, the presence of phenols, specifically butylated hydroxytoluene (BHT, P28) in the current study, as potentially characteristic of all Egyptian cvs to account for 55.1, 37.2, and 77.2% in E-SFG, E-RG-W, and E-RG, respectively, is first time to be reported, Table 2. BHT is a classical antioxidant commonly used as a reference standard in assessment of the antioxidant activity of natural products and in food industry beside to butylated hydroxyanisole (BHA)^36^. Nevertheless, it has been reported previously in the fruit's pericarp of litchi (*Litchi chinensis* Sonn.)^37^. This finding is likely to potentiate the antioxidant activity of guava fruits which are naturally rich in vitamin C (ascorbic acid) even than citrus fruits, i.e., orange^4,38^. These results ought to be further pursued by analysing guava fruits from other origins to confirm BHT abundance ideally using other spectrometric tools such as NMR especially considering its potential health benefits.

### Aliphatic hydrocarbons

Five peaks were identified as aliphatic hydrocarbons in guava cvs. ranging from 7.3 to 30.1% of total VOCs, Table 2. The Egyptian cvs. showed the highest levels attributed mostly to 2,4-dimethyl decane (P20) that was apparent clearly in the sample harvested in winter, i.e., E-RG-W detected at 30.1%. 2,4-Dimethyl decane (P20) and dodecane (P13) were considered the most predominant accounting for 20.0–10.2% for E-RG-W, and 16.5–9.8% in E-SFG, respectively. These results confirmed that volatiles profile in winter differed from that harvested in summer as observed in sesquiterpene hydrocarbons. With regards to cvs., lower abundances of aliphatic hydrocarbons were observed comparable to that in Egyptian E-RG at ca. 7%., where 2,3-dimethyl-1,3-butadiene (P5) was the most abundant in both Indian cvs., Table 2. These findings proved additionally that the geographical origin affected VOCs profile of guava fruits as observed in other fruits aroma such as mango and dates^22,39^.

### Sulphur compounds

Despite only that 2 VOCs were identified in guava fruits belonging to sulphur compounds represented by 3-butenyl isothiocyanate (P9) and 2-(allylthio) acetonitrile (P15), they showed potential differences among Egyptian vs. Indian guava fruits. Sulphur compounds were found nearly absent in Egyptian cvs., albeit detected in Indian derived fruits at 14 and 8.7% for I-RG-R and I-RG-W, respectively, Table 2. Consequently, both sulphur compounds may be also used as potential markers for discrimination guava cvs. of Indian origin from that of Egypt.

### Alcohols and aldehydes

Alcohols and aldehydes were represented by 4 compounds of which their levels seem to be affected by fruit origins as observed in case of sulphur compounds, their abundances. Higher levels were detected in Indian guava compared with Egyptian cvs., at which they were detected at trace levels, Table 2. The red cv. (I-RG-R) was more abundant in alcohols (19.2 in red vs. 15.8% in white), whereas white (I-RG-W) was richer in aldehydes at 5.5% in white vs. 3.9% in red. The higher alcohols level in I-RG-R was attributed to 3-hexen-1-ol (P4) and muurolol (P38) accounting for 8.3% and 6.9%, respectively. Moreover, hexanal (P1) and (*E*)-2-hexenal (P2) were major aldehydes in I-RG-W at 3.1 and 1.8%, respectively. Such aldehyde levels were considered though much lower in comparison with previous studies to reach 80% in white flesh guava cv. grown in Reunion Island and red guava from Dominican Republic^33,40^.

### Miscellaneous

Other VOCs classes were identified included monoterpene hydrocarbons, ketones/esters, and nitrogenous compounds detected at 1.2–3.6%, 0.2–2.3%, and 0.02–1.5%, respectively. Among monoterpene hydrocarbons, *β*-myrcene (P8) was the major form accounting for 3.5% in E-RG-W, 6-methyl-5-hepten-2-one (P10) as a ketone represents 1.9% in I-RG-W, and 3-phenyl propiononitrile (P17) as nitrogenous compound detected at ca. 1.3% in I-RG-R, Table 2.

### Multivariate data analyses of guava fruit cvs. aroma composition

Despite that clear differences in aroma composition could be observed by visual inspection of GC–MS chromatograms Fig. 1, MVDA was applied to aid in cvs. discrimination and identification of potential markers in an untargeted manner. The untargeted or unsupervised modeling (e.g., PCA) was applied, and then the targeted or supervised (e.g., OPLS-DA) approach to validate findings from PCA model. In the following subsections, both modeling approaches shall be discussed alongside their findings and comparison among their results.

#### Unsupervised PCA and HCA data analyses of guava fruit cvs.

**Figure 3:**
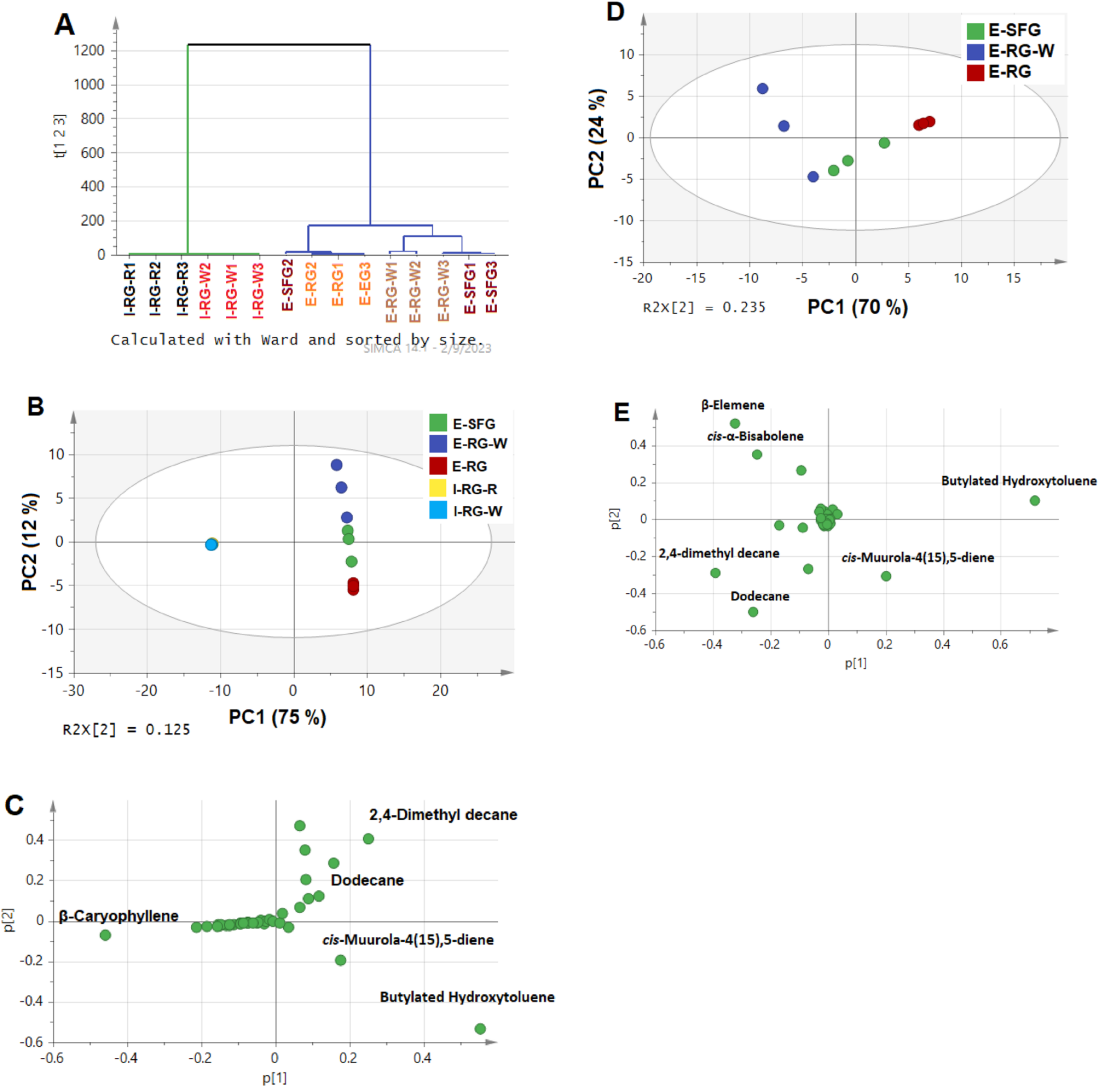
Unsupervised analysis of all investigated guava fruits collected from Egypt and India based on identified volatiles, including (**A**) HCA, (**B**) PCA score plot, and (**C**) PCA loading plot. In addition, Egyptian samples were analysed using PCA in (**D**) score plot and (**E**) loading plot. The sample codes are listed in Table 1.

The parameters of model fitting were assessed firstly based on covered variation (R2X) and predicted variation (Q2) for the first two components showing values at 0.87 and 0.72, respectively. Besides, both HCA and PCA score plot in Fig. 3A and B, respectively, demonstrated that the three biological replicates (n = 3) of each sample were found to be reproducible, reflected by the more or less superimposable scores of replicate measurements and sub-clusters of HCA model.

The HCA model (Fig. 3A) could separate guava cvs. into 2 main clusters. The 2 clusters seemed to be related to geographical origin, in which Indian clustered in the negative left side together away from Egyptian fruits. Additionally, PCA score plot (Fig. 3B) confirmed the previous finding of HCA, where the Egyptian guava cvs. segregated clearly away from Indian counterparts mainly along PC1. The PC1 accounted for 75% of the total variance (87%), proving the potential power of constructed model in analyzing variance among fruits. Moreover, Indian cvs. were superimposable and could not be differentiated, while the Egyptian cvs. could be discriminated along PC2 accounting though for only12% of the total variance. It is also interesting to note that winter collected sample E-RG-W was clustered mainly in the upper quarter of the positive side of PCA score plot model. This result may reveal further discrimination and prove the previous findings of volatiles analysis in Table 2. Furthermore, to aid identifying main markers responsible for such classification and discrimination, PCA loading plot was examined (Fig. 3C) revealing that *β*-caryophyllene (P30) predominated mostly in Indian cvs., while 2,4-dimethyl decane (P20) and BHT (P28) were characteristic for Egyptian cvs., particularly E-RG-W and E-RG, respectively.

#### Unsupervised PCA of Egyptian guava fruit cvs.

Further analyses of Egyptian cvs. was attempted by modelling them alone. The model fitting parameters, i.e., R2X and Q2, recorded more significant values reaching 0.94 and 0.82, respectively. The PCA score plot (Fig. 3D) was able to discriminate samples along PC1 that accounted for 70% of the total variance (94%). The replicates of each sample were located in 3 different zones, where the winter sample showing rough skin (E-RG-W) was allocated mainly in the left upper side with negative score values opposite to summer sample characterized by rough skin with yellowish white pulp (E-RG), whereas third sample harvested in summer with soft skin and yellowish white pulp (E-SFG) was in between. Interestingly, loading plot (Fig. 3E) revealed a number of potential markers such as *β*-elemene (P21), *cis*-α-bisabolene (P36), and 2,4-dimethyl decane (P20), and dodecane (P13) which were more abundant in E-RG-W, versus BHT (P28) in E-RG, and *cis*-muurola-4(15),5-diene (P34) in E-SFG. These findings fall in agreement with the previous results discussed in aroma profiles analysis.

#### Supervised OPLS-DA of Indian guava fruit cvs. aroma profile

Likewise, Indian guava cvs. aroma profile was modelled separately using supervised data analyses to minimize variance helping for enhancing samples classification and discrimination between specimens^41^. Since, the untargeted modelling could not differentiate between both Indian cvs. as shown previously in Fig. 3B, they were modelled alternatively with this supervised tool. The model fitted well showing values for a goodness of fit (R^2^) and prediction ability (Q^2^) at 0.99 and 0.98. respectively.

**Figure 4:**
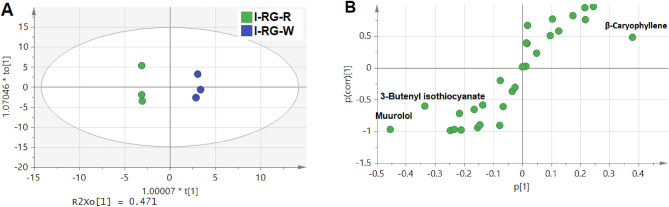
Supervised OPLS-DA analysis of Indian guava fruit samples based on volatiles using (**A**) score plot and (**B**) loading plot. The samples code are listed in Table 1.

The OPLS-DA score plot showed good segregation of replicates belonged to both cvs., where the I-RG-W and I-RG-R aligned in the positive and negative side, respectively, Fig. 4A. Additionally, loading plot revealed that *β*-caryophyllene (P30), 3-butenyl isothiocyanate (P9), and muurolol (P38) were found to be potential markers, in which the first volatile (P30) was more correlated with the white pulp fruit cv. (I-RG-W), Fig. 4B.

### Changes in gauva fruits juice aroma composition with probiotics

*Lactobacillus plantarum* and *Lactobacillus acidophilus* are good candidates for investigating probiotics preparation as food source, since they are probiotic bacteria that inhabit human gut symbiotically^42,43^. Guava fruit constitutes a nutritional and flavoring agent in several fermented dairy products, i.e., acidophilus milk and yogurt, change in its aroma composition is thus expected upon incubation with probiotics and has never been examined in literature.

Similarly, unsupervised and supervised modeling were performed to assess for changes in guava aroma with probiotics inoculation. The aroma changes in response to *L. acidophilus* (Fig. S2A–C) and *L. plantarum* (Fig. S2D–F) is shown at 3 different time points, i.e., 0, 24, and 48 h. However, both organisms resulted in no clear segregation in PCA score plot between the different 3-time intervals (Fig. S2A,D) with a negative prediction power (Q^2^). Hence, supervised OPLS-DA modeling was further applied for better classification in Fig. S2B and E. The latter showed score plot models with slightly separated replicates along PC1, where the aroma profile at 48 h differed from the other 2-time intervals and appeared at the left negative side. Nevertheless, validation of the models through permutation test demonstrated a non-significant model with *p* > 0.05, Fig. S2C and F. Modelling results proved that guava fruit juice aroma is unlikely to be affected by probiotics and preserve its unique characteristics in contrast to mango puree^22^.

Furthermore, OPLS-DA loading plot for the Egyptian guava fruits following probiotic inoculation is shown in Fig. S3. Results showed that *L. acidophilus* led to increase in *β*-caryophyllene and *α*-cadinol (Fig. S3A), while *L. plantarum* was more linked to increase in nonadecane (Fig. S3B) at 48 h.

## Conclusion

Guava fruit is a chief constituent in various food recipes, including desserts, fermented dairy products, juices, jams, and others owing to its rich nutritional composition. Its flavor is also attractive for consumers, mostly attributed to its unique volatiles profile. Considering the different cvs. for this fruit and its inclusion in several probiotic based food, a comprehensive investigation of its aroma was undertaken. The current study represents the first comparative aroma study of various guava cvs. of different origin (tropical versus subtropical) and types. Overall, aroma profiles showed predominance of sesquiterpenes, aliphatic hydrocarbons, and monoterpene hydrocarbons in agreement with other studies. Nevertheless, results showed that the geographical origin, season of harvesting, and color of fleshy pulp were determinant factors in aroma composition. For instance, Egyptian grown guava fruits were substantially different from Indian fruits, particularly being rich in BHT and less sesquiterpene hydrocarbons. In contrast, Indian grown cvs. were more abundant in sesquiterpene hydrocarbons, sulphur compounds, and alcohols. Also, fruit harvested in winter was found to be different from summer in same location as manifested by differences in aliphatic and monoterpene hydrocarbons. Particularly, *β*-caryophyllene was a potential marker discriminating the Indian from Egyptian cvs. However, probiotic treatment was non-significant without influencing aroma profile or production of novel volatiles. Further analytical tools should follow now exploring non-volatile constituents, including techniques such as liquid chromatography coupled to mass spectroscopy (LC–MS) and NMR.

### Supplementary Information

Supplementary Figures.

## Data Availability

The datasets analyzed during the current study are available from the corresponding author upon reasonable request.
