# Supplementary material for: Aroma-based discrimination of Egyptian versus Indian guava fruits and in response to probiotics as analyzed via SPME/GC–MS and chemometric tools
Source: Sci Rep. 2023 Oct 27;13:18420. doi: 10.1038/s41598-023-45686-z (PMC10611719; doi:10.1038/s41598-023-45686-z)
Supplement: Supplementary file 1 — Supplementary Figures. [file 41598_2023_45686_MOESM1_ESM.docx]

**Aroma-based Discrimination of Egyptian versus Indian Guava Fruits and in Response to Probiotics as Analyzed *via* SPME/GC-MS and Chemometric Tools**

Islam M. Kamal**^a1^**, Ahmed Zayed**^b1^** , Tarek F. Eissa**^c^**, Mohamed A. Farag**^d*^**

**^a^** Microbiology and Immunology Department, Faculty of Pharmacy, Cairo University, Kasr El Aini, 11562 Cairo, Egypt

**^b^** Pharmacognosy Department, College of Pharmacy, Tanta University, Elguish street (Medical Campus), 31527 Tanta, Egypt

**^c^** Pharmacognosy Department, College of Pharmacy, Modern Science and Arts University, Cairo 12566, Egypt

**^d^** Pharmacognosy Department, College of Pharmacy, Cairo University, Kasr El Aini St., P.B. 11562 Cairo, Egypt

**^1^** Equal contribution

**^*^Correspondence**

**Prof. Dr. Mohamed A. Farag**

Cairo University, College of Pharmacy, Department of Pharmacognosy, Egypt.

**E-mail addresses:** mohamed.farag@pharma.cu.edu.eg

**Tel:** +011-202-2362245

**Fax:** +011-202-25320005

**Supplementary information**


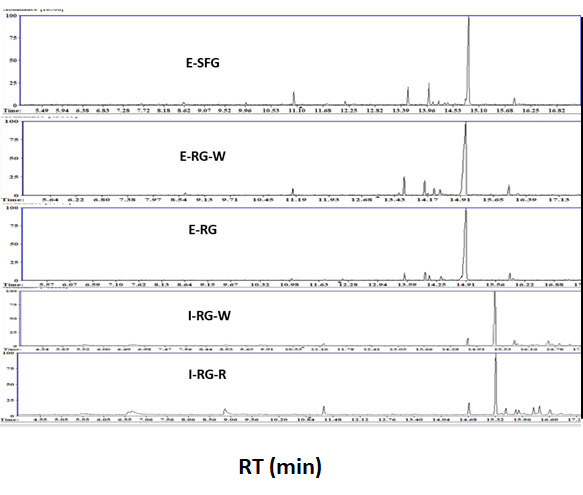


**Figure S1**: GC-MS chromatograms of investigated guava fruits for their volatiles analysed by SPME coupled with GC-MS. The samples codes are listed in **Table 1**.

| 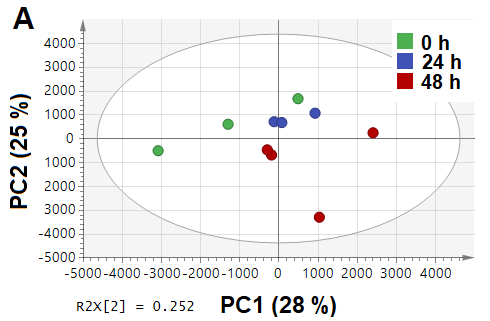 | 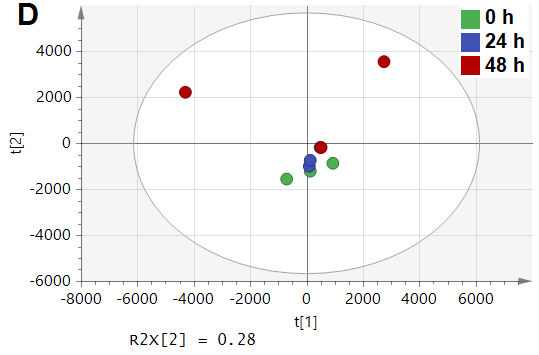 |
| --- | --- |
| 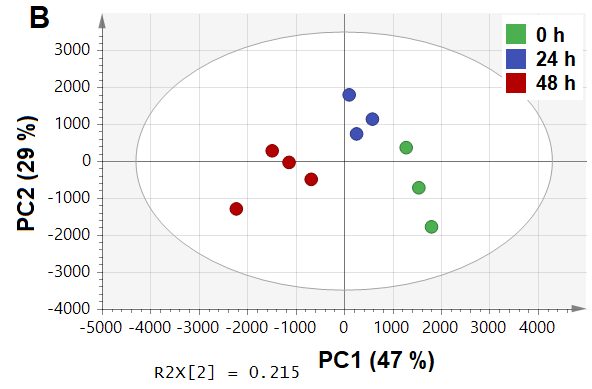 | 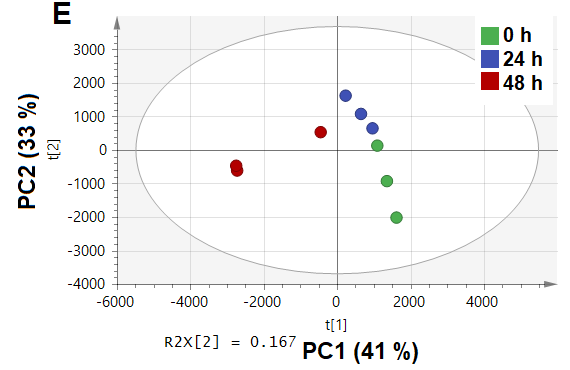 |
| 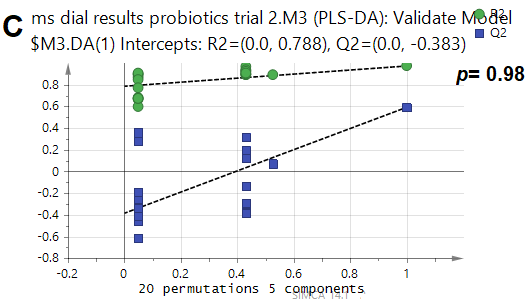 | 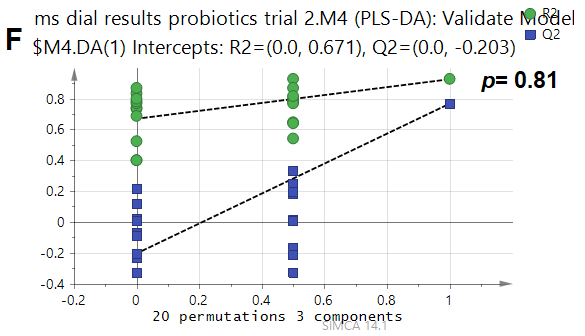 |

**Figure S2**: Chemometrics (unsupervised and supervised) analyses of the Egyptian guava fruit volatiles following probiotic fermentation using *Lactobacillus acidophilus* (A-C) and *L. plantarum* (D-F) at 3 different time intervals, i.e., 0, 24, and 48 h. A and D represent the PCA score plot, B and E are OPLS-DA score plot, while C and F are models’ validation by 20 permutations test and respective p-value estimation.

**
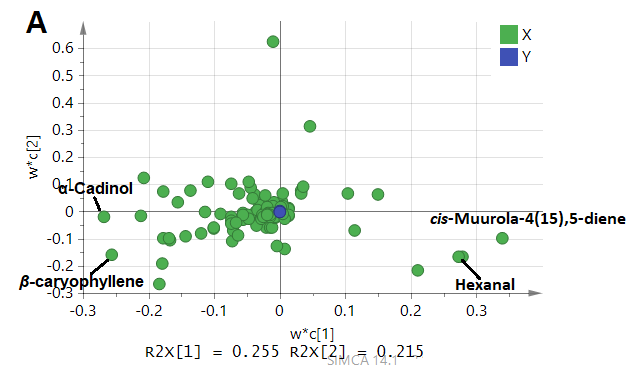

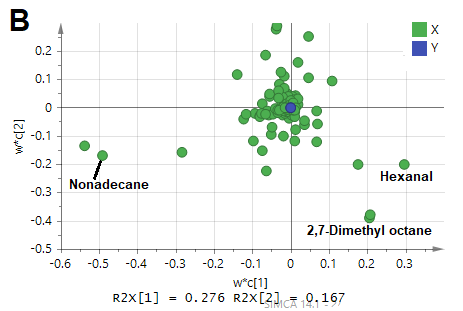
**

**Figure S3**: OPLS-DA loading plot for Egyptian guava fruits following probiotic fermentation by A) *Lactobacillus acidophilus* and B) *L. plantarum* based on identified volatiles.
